# Neuroprotective Effects of a Novel Single Compound 1-Methoxyoctadecan-1-ol Isolated from *Uncaria sinensis* in Primary Cortical Neurons and a Photothrombotic Ischemia Model

**DOI:** 10.1371/journal.pone.0085322

**Published:** 2014-01-08

**Authors:** Ji Yeon Jang, Young Whan Choi, Ha Neui Kim, Yu Ri Kim, Jin Woo Hong, Dong Won Bae, Se Jin Park, Hwa Kyoung Shin, Byung Tae Choi

**Affiliations:** 1 Division of Meridian and Structural Medicine, School of Korean Medicine, Pusan National University, Yangsan, Gyeongnam, Republic of Korea; 2 Department of Horticultural Bioscience, College of Natural Resource and Life Science, Pusan National University, Miryang, Gyeongnam, Republic of Korea; 3 Department of Korean Medical Science, School of Korean Medicine, Pusan National University, Yangsan, Gyeongnam, Republic of Korea; 4 Division of Clinical Medicine 1, School of Korean Medicine, Pusan National University, Yangsan, Gyeongnam, Republic of Korea; 5 Central Instrument Facility, Biomaterial Analytical Lab., Gyeongsang National University, Jinju, Gyeongnam, Republic of Korea; University of Texas Medical Branch, United States of America

## Abstract

We identified a novel neuroprotective compound, 1-methoxyoctadecan-1-ol, from *Uncaria sinensis* (Oliv.) Havil and investigated its effects and mechanisms in primary cortical neurons and in a photothrombotic ischemic model. In primary rat cortical neurons against glutamate-induced neurotoxicity, pretreatment with 1-methoxyoctadecan-1-ol resulted in significantly reduced neuronal death in a dose-dependent manner. In addition, treatment with 1-methoxyoctadecan-1-ol resulted in decreased neuronal apoptotic death, as assessed by nuclear morphological approaches. To clarify the neuroprotective mechanism of 1-methoxyoctadecan-1-ol, we explored the downstream signaling pathways of N-methyl-D-aspartate receptor (NMDAR) with calpain activation. Treatment with glutamate leads to early activation of NMDAR, which in turn leads to calpain-mediated cleavage of striatal-enriched protein tyrosine phosphatase (STEP) and subsequent activation of p38 mitogen activated protein kinase (MAPK). However, pretreatment with 1-methoxyoctadecan-1-ol resulted in significantly attenuated activation of GluN2B-NMDAR and a decrease in calpain-mediated STEP cleavage, leading to subsequent attenuation of p38 MAPK activation. We confirmed the critical role of p38 MAPK in neuroprotective effects of 1-methoxyoctadecan-1-ol using specific inhibitor SB203580. In the photothrombotic ischemic injury in mice, treatment with 1-methoxyoctadecan-1-ol resulted in significantly reduced infarct volume, edema size, and improved neurological function. 1-methoxyoctadecan-1-ol effectively prevents cerebral ischemic damage through down-regulation of calpain-mediated STEP cleavage and activation of p38 MAPK. These results suggest that 1-methoxyoctadecan-1-ol showed neuroprotective effects through down-regulation of calpain-mediated STEP cleavage with activation of GluN2B-NMDAR, and subsequent alleviation of p38 MAPK activation. In addition, 1-methoxyoctadecan-1-ol might be a useful therapeutic agent for brain disorder such as ischemic stroke.

## Introduction

The hooks and stems of dried *Uncaria sinensis* (Oliv.) Havil have been widely used in traditional Korean medicine as a pharmacological medicine against diverse neurological symptoms. *U. sinensis* contain various classes of bioactive compounds, including organic acid (caffeic acid), flavonoid (catechin and epicatechin), and alkaloid (rhynchophylline) [1]–[3]. Rhynchophylline has been studied intensively as a main alkaloid constituent of *Uncaria* species and exhibits antihypertension and neuroprotection activities linkage with traditional concept and uses [4].

Our previous results demonstrated that a hexane extract from *U. sinensis* protects against cerebral ischemic damage through regulation of Akt/endothelial nitric oxide synthase signaling [5] and exerts significant anti-apoptotic effects against glutamate-induced neurotoxicity [6]. Because it has not been determined which compounds of *U. sinensis* are bioactive, we isolated a novel single compound, 1-methoxyoctadecan-1-ol, from specific fractions of hexane extracts using an neuronal cells and ischemic animal model for screening of active constituents.

Excitotoxic neuronal death via over-activation of the N-methyl-D-aspartate receptor (NMDAR) contributes to excessive Ca^2+^ uptake and promotes activation of calcium-activated proteases, including calpain and caspases; these proteases act as a signal, triggering the cell death signaling pathway [7]–[9]. Recent studies have suggested that toxic glutamate or excessive activation of NMDAR can activate neuronal proteases such as calpain and subsequently triggers calpain-mediated cleavage of striatal-enriched tyrosine phosphatase (STEP), which may contribute to neurotoxicity [10], [11].

STEP, a brain-specific tyrosine phosphatase, is thought to have critical roles in normal synaptic function and pathological states [12]. Recent findings have demonstrated that conversion of STEP_61_ to STEP_33_ by activated calpain following excitotoxic or ischemic insult results in a change in function, and this change in STEP results in activation of p38 MAPK, leading to initiation of cell death signaling pathways [10], [12], [13]. Treatments preventing cleavage of STEP may be useful therapeutic strategies against glutamate excitotoxicity and oxygen-glucose deprivation models in stroke/ischemia [12], [14]–[16].

The protective effects of *U. sinensis* extract and its phenolic and alkaloid constituents on glutamate or ischemic neurotoxicity were reported [1], [17], [18], however, detailed mechanisms of neuroprotection in control of calpain-mediated neurons specific tyrosine phosphatase STEP signaling with NMDAR remain unclear. Therefore, we explored the neuroprotective effects of 1-methoxyoctadecan-1-ol against glutamate-induced neurotoxicity and photothrombotic cortical ischemia and attempted to clarify its relative mechanism for neuronal death. The current study provides a first evaluation of the neuroprotective effects of 1-methoxyoctadecan-1-ol focused on excitotoxicity with ischemic insult.

## Materials and Methods

### Preparation of 1-methoxyoctadecan-1-ol

Dried hooks and stems of *U. sinensis* (2.02 kg) were ground to a fine powder and then successively extracted at room temperature with n-hexane (14.53 g), ethylacetate (21.36 g), and methanol (MeOH, 63.32 g). The hexane extract (11.31 g) was evaporated in vacuo and chromatographed on a 40 µm silica gel column (100 cm×3.5 cm; Baker, Phillipsburg, NJ, USA) with a step gradient of 50% methylene chloride (CH_2_Cl_2_) in hexane, 5, 20% acetone in CH_2_Cl_2_, and 5, 25, and 50% MeOH in CH_2_Cl_2_ to obtain 62 fractions. Fraction 6 (JGH6-7, 414.1 mg) was separated on a Sephadex column (78 cm×3.0 cm, Sigma–Aldrich, St. Louis, MO, USA) with a 50∶50 mixture of CHCl3 and MeOH to obtain three fractions. Fraction 2 (JCKH6IB, 206.1 mg) was separated on a silica gel column (100 cm×3.0 cm, Baker) with a 50∶50 mixture of hexane and CH_2_Cl_2_ to yield 1-methoxyoctadecan-1-ol (37.2 mg). Purified 1-methoxyoctadecan-1-ol was identified by high-pressure liquid chromatography on a ZORBAX Eclipse Plus C18 column (250 mm×4.6 mm ID, 5 µm particle size; Agilent Technologies, Santa Clara, CA, USA) with a methanol–acetonitrile gradient, at a flow rate of 1.0 ml/min, using ultraviolet detection at a wavelength of 254 nm.

### Chemicals and Antibodies

Calpeptin was purchased from Calbiochem (Cambridge, MA, USA). Cytosine arabinoside, Hochest 33342, L-glutamate, 3-(4,5-dimetylthiazol-2-yl)-2,5-diphenyl tetrazolium bromide (MTT), poly-L-lysine, SB203580, and β-actin antibody were purchased from Sigma-Aldrich (St. Louis, MO, USA). All neuronal cell culture media and chemicals were purchased from Gibco/Invitrogen (Carlsbad, CA, USA). For our investigations, the following antibodies were used: α-fodrin, CREB, ERK, p38, calpain1, calpain2, phospho-CREB (pCREB, Ser133), phospho-ERK (pERK, Thr202), phospho-p38 (pp38, Thr180/Tyr182), and phospho-GluN2A (pGluN2A, Tyr1246) antibodies were purchased from Cell Signaling Technology (Danvers, MA, USA). GluN2A, GluN2B, and phospho-GluA1 (pGluA1, Ser845) antibodies were purchased from Millipore (Billerica, MA, USA). GluA1, GluA2, and phospho-GluA2 (pGluA2, Ser880) antibodies were purchased from Abcam (Cambridge, UK). Phospho-GluN2B (pGluN2B, Ser1303) antibody was purchased from Upstate Biotechnology (Lake Placid, NY, USA). Mouse monoclonal STEP antibody was purchased from Novus Biologicals (Littleton, CO, USA). Secondary antibodies were purchased from Santa Cruz Biotechnology (Santa Cruz, CA, USA). A FITC Annexin V apoptosis detection kit was purchased from BD Bioscience (San Diego, CA, USA). A lactate dehydrogenase (LDH) cytotoxicity assay kit and terminal deoxynucleotidyl transferase-mediated dUTP nick end labeling (TUNEL) assay kit were purchased from Promega (Madison, WI, USA).

### Primary Rat Cortical Culture

Primary rat cortical neuronal cultures were obtained from the brains of embryonic day 17–18 Sprague-Dawley rat fetuses (Dooyeol Biotech, Seoul, Korea). All procedures used in these studies followed the guidelines of protocols approved by the Pusan National University Animal Care and Use Committee in accordance with the National Institutes of Health Guidelines (Permit Number: PNU-2011-000411). Cortices were dissected from embryonic brain, and the tissue was dissociated by tyrpsinization and re-suspended in DMEM supplemented with 10% FBS, 1 mM pyruvate, 4.2 mM sodium bicarbonate, 20 mM HEPES, 0.3 g/l bovine serum albumin, and 1% penicillin/streptomycin. Cells were plated at a density of 1×10^6^ cells/ml per tissue culture dish pre-coated with poly-L-lysine in a 5% humidified CO_2_ incubator at 37°C. Forty eight hours later, cytosine arabinoside (5 µM) was added in order to inhibit proliferation of non-neuronal cells. At three days after plating, the medium was changed to neurobasal medium containing 2% B27 supplement, 0.5 mM glutamine, and 1% penicillin/streptomycin. All experiments were performed on neurons grown for 9–10 days in vitro.

### MTT Assay

Neuronal cell survival was assessed using an MTT assay. The culture medium was removed and replaced with 0.5 mg/ml MTT solution, followed by incubation for 4 h at 37°C. At the end of treatment, MTT medium was carefully removed and blue formazan dye was solubilized using dimethyl sulfoxide. Optical density was measured at 570 nm using a Spectra MAX 190 spectrometer (Molecular Devices, Sunnyvale, CA, USA). Results were expressed as a percentage of control.

### LDH Assay

Cytotoxicity was evaluated by measuring the release of LDH in culture medium by dead cells. The treated neurons were lysed with 0.1% Triton X-100 for 45–60 min at 37°C to induce maximal cell lysis. Each supernatant sample was subsequently incubated with substrate for 30 min at room temperature protected from light. To stop the reaction, a stop solution was added and absorbance was measured at 490 nm using a Spectra MAX 190 spectrometer (Molecular Devices). Data represent the percentage of LDH released relative to controls.

### Hoechst 33342 Staining

Neurons were grown on poly-L-lysine-coated chamber slides (Thermo Fisher Scientific Inc. Rochester, NY, USA) at a density of 1×10^5^ cells/ml. The treated neurons were washed with phosphate-buffered saline and fixed in 4% paraformalehyde for 20 min at 4°C. Fixed cells were washed three times with PBS and stained with 10 µg/ml of Hoechst 33342 in PBS for 15 min at 37°C. The cells were washed three times with PBS and mounted using the medium for fluorescence (Vector Laboratories, Inc.). The morphology of viable and dead cells was visualized using a fluorescence microscope (Carl Zeiss, Gottingen, Germany). Apoptotic cells were defined as neurons with fragmented or condensed DNA. Data are presented as the ratio of chromosomal condensation and morphological change as a percentage of total cells.

### TUNEL Assay

Neurons were grown on poly-L-lysine-coated chamber slides (Thermo Fisher Scientific Inc.) at a density of 1×10^6^ cells/ml. The treated neurons were fixed in 4% formaldehyde for 25 min at 4°C and cells were permeabilized using 0.2% Triton X-100 solution in PBS for 5 min. Next, cells were incubated with nucleotide mix and TdT (Terminal deoxynucleotidyl Transferase) enzyme for 60 min at 37°C. At the end of the reaction, the DNA-labeling reaction was terminated by addition of 2X SSC and washing with PBS, followed by counterstaining with 1 µg/ml of propidium iodide (PI) in PBS for 15 min at room temperature in the dark. Apoptotic neurons were determined by localization of green fluorescent cells (flurescencein-12-dUTP) on a red background using a fluorescence microscope (Carl Zeiss). Data are presented as apoptotic cells as a percentage of total cells.

### Flow Cytometry Analysis

The treated neurons were harvested and re-suspended in binding buffer at a concentration of 1×10^5^ cells/ml, followed by addition of 5 µl of Annexin V-FITC and 5 µl of PI solution and incubated for 15 min at room temperature. At the end of the reaction, 400 µl of binding buffer was added, followed by analysis using flow cytometry (FACS CantoTM II; Becton Dickinson, San Jose, CA, USA).

### Western Blotting

Samples were lysed with lysis buffer (200 mM Tris [pH8.0], 150 mM NaCl, 2 mM EDTA, 1 mM NaF, 1% NP40, 1 mM PMSF, 1 mM Na_3_VO_4_, and Protease inhibitor cocktail). The total protein concentrations of the lysates were determined using the Bradford protein assay and 30 µg of protein was separated by 10∼12% SDS-polyacrylamide gel electrophoresis and then transferred to a membrane. After transfer, membranes were incubated with 5% skim milk in PBST for 1 h, followed by incubation overnight at 4°C with the primary antibodies. The blots were washed and incubated with appropriate HRP-conjugated secondary antibodies (Santa Cruz Biotechnology) for 1 h. All bands were detected using an ECL detection kit (Pierce Biotech, Rockford, IL, USA), and imaged using an Image Quant LAS-4000 image system (GE healthcare Life Science, Uppsala, Sweden). Results of Western blot assay reported here are representative of at least three experiments.

### Photothrombotic Cortical Ischemia

Male C57BL/6J mice (20–25 g) were used for study. All animals were housed under diurnal lighting with 12 h cycles of dark and light, and fed with a standard rodent diet *ad libitum*. For surgical procedures, animals were anesthetized using 1% isoflurane in air; after a middle scalp incision and pericranial tissue dissection, bregma and lambda points were identified. A fiber optic bundle of a cold light source (KL1500 LCD, Carl Zeiss, Gottingen, Germany) with a 4 mm aperture was centered using a micromanipulator at 2 mm laterally from bregma. Rose Bengal (Sigma–Aldrich), a photosensitive dye, was dissolved in sterile saline at a concentration of 10 mg/ml, and 1 mg of Rose Bengal in 0.1 ml was injected intraperitoneally (i.p) 5 min before illumination. The brain was illuminated through the intact skull for 15 min. All mice tolerated the entire procedure very well, showed behavioral deficits, and survived from brain ischemia.

### Infarct Volumes and Neurological Deficits

Brains were removed 24 h after ischemia insults. Cerebral infarct size was determined on 2,3,5-triphenyltetrazolium chloride (TTC)-stained, 2 mm-thick brain sections. Areas of infarction were quantified using iSolution full image analysis software (Image & Microscope Technology, Vancouver, Canada). To account for and eliminate the effects of swelling/edema, infarction volume was calculated using an indirect measurement by summing the volumes of each section according to the following formula: contralateral hemisphere (mm^3^) – undamaged ipsilateral hemisphere (mm^3^). Edema volume was calculated as: damaged ipsilateral hemisphere (mm^3^) - indirect infarct volume (mm^3^). After 24 h of ischemic insult, the mice were evaluated using a five-point neurological deficit score; grade 0, no deficit; 1, forelimb weakness and torso turning to the ipsilateral side when held by the tail; 2, circling to the affected side; 3, unable to bear weight on the affected side; and 4, no spontaneous locomotor activity or barrel rolling.

### Data Analysis

All data were expressed as mean±SEM and were analyzed using the Sigmastat statistical program version 11.2 (Systat Software, San Jose, CA, USA). Statistical comparisons were performed using paired or unpaired Student’s t-test and one-way analysis of variance (ANOVA) or two-way ANOVA for repeated measures followed by Fisher’s protected least significant difference test. A value of *p*<0.05 was considered statistically significant.

## Results

### Determination of the Structure of Active 1-methoxyoctadecan-1-ol

**Figure 1:**
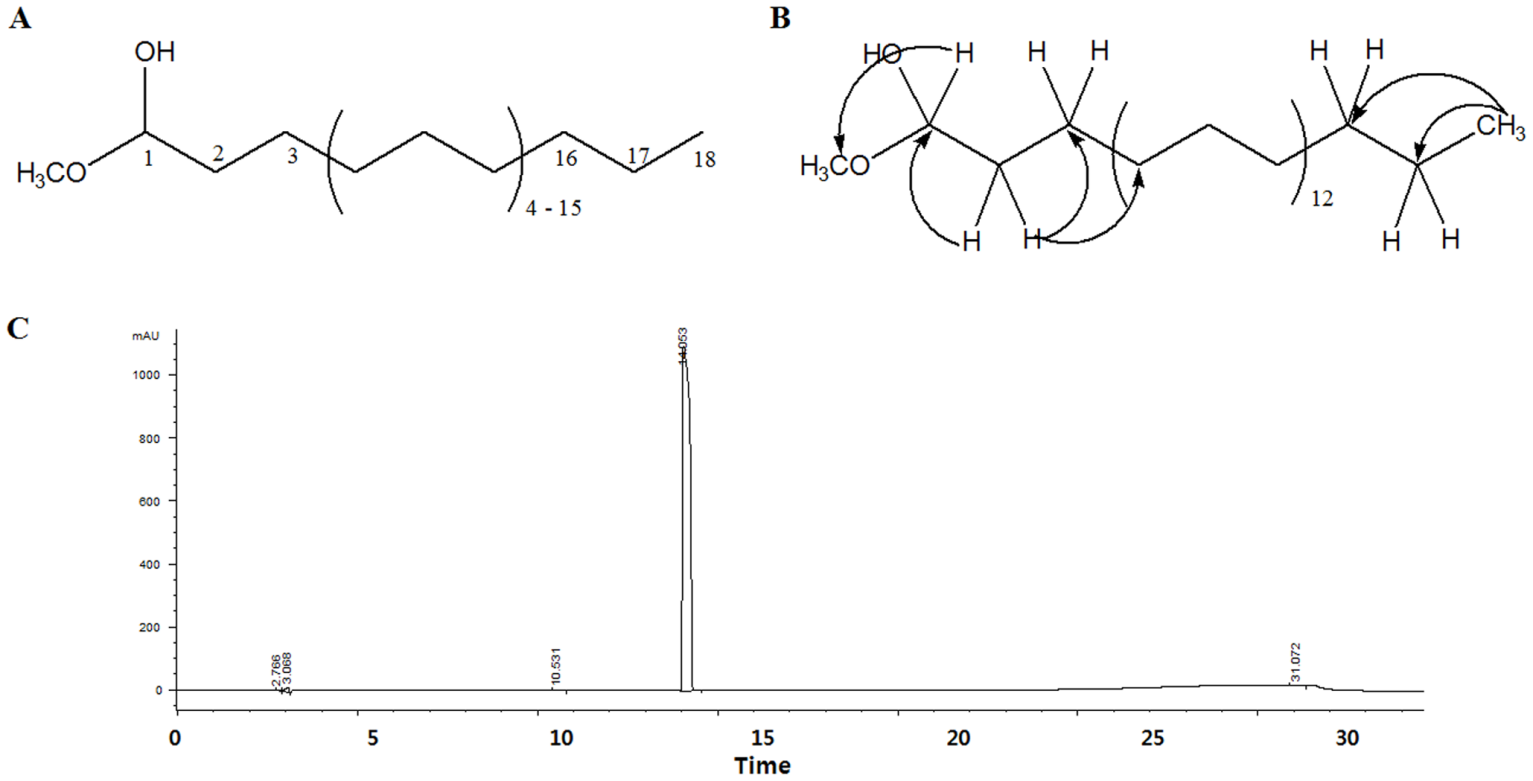
Identification of 1-methoxyoctadecan-1-ol from *U. sinensis*. Structure of 1-methoxyoctadecan-1-ol (A). Key HMBC correlations of 1-methoxyoctadecan-1-ol (B). HPLC profile showed that the active compound had a purity of >96% (C).

The active molecule isolated from *U. sinensis* was identified by ^1^H and ^13^C distortionless enhancement by polarization transfer (Dept), heteronuclear single quantum coherence (HSQC), and heteronuclear multiple bond correlation (HMBC) nuclear magnetic resonance (NMR) spectra in chloroform D (CDCl_3_). The 1-methoxyoctadecan-1-ol was obtained as a white sticky residue with [α]_21_^D^ −45.1 (ca. 0.35, CHCl_3_). From the molecular ion signal in liquid chromatography–mass spectrometry at m/z 301 [M+1]^+^ of 1-methoxyoctadecan-1-ol, an elemental composition of C_19_H_40_O_2_ was ascertained. This finding was confirmed using the ^1^H, ^13^C, and Dept NMR technique, which showed two primary (δC 52.80, 14.34), 16 secondary (δC 32.73, 32.16, 29.94×6, 29.91, 29.89, 29.88, 29.80, 29.72, 29.60, 24.84, 22.93), and one tertiary (δC 104.82). As indicated by the elemental composition of C_19_H_40_O_2_, the aliphatic system must be present. ^1^H NMR of 1-methoxyoctadecan-1-ol show edone methoxyl singlet (δH 3.31, OCH_3_, s) and methylene groups at δH  = 2.16 (1-H, d, J  = 12.0), 3.53 (4-H–18-H, m, mix of H-4 to H18), 1.59 (2-H, m), and 1.30 (3-H, m). Absorption of one of the methine groups was detected at δH  = 4.36 (1-H, t) (Table 1). A purity of 1-methoxyoctadecan-1-ol from *U. sinensis* showed more than 96% purity (Fig. 1).

**Table 1:** ^1^H (500 MHz in CDCl_3_), ^13^C NMR (125 MHz in CDCl_3_) data and key HMBC correlations of 1-methoxyoctadecan-1-ol.

No	δ_C_		δ_H_	HMBC
1	104.82	CH	4.36, t	52.80, 32.73
2	32.73	CH_2_	1.59, m	104.82, 24.84, 29.??
3	24.84	CH_2_	1.30, m	
4–16	29.60	CH_2_	1.26	
	29.72	CH_2_	1.26	
	29.80	CH_2_	1.26	
	29.88	CH_2_	1.26	
	29.89	CH_2_	1.26	
	29.91	CH_2_	1.26	
	29.94×6	CH_2_	1.26	
17	32.16	CH_2_	1.26, m	
18	22.93	CH_2_	1.26 m	
19	14.34	CH_3_	1.88, t	32.16, 22.93
OCH_3_	52.80	CH_3_	3.31, s	104.82

### 1-methoxyoctadecan-1-ol Treatment Elicits a Concentration-dependent Neuroprotective Effect against Glutamate-induced Toxicity

**Figure 2:**
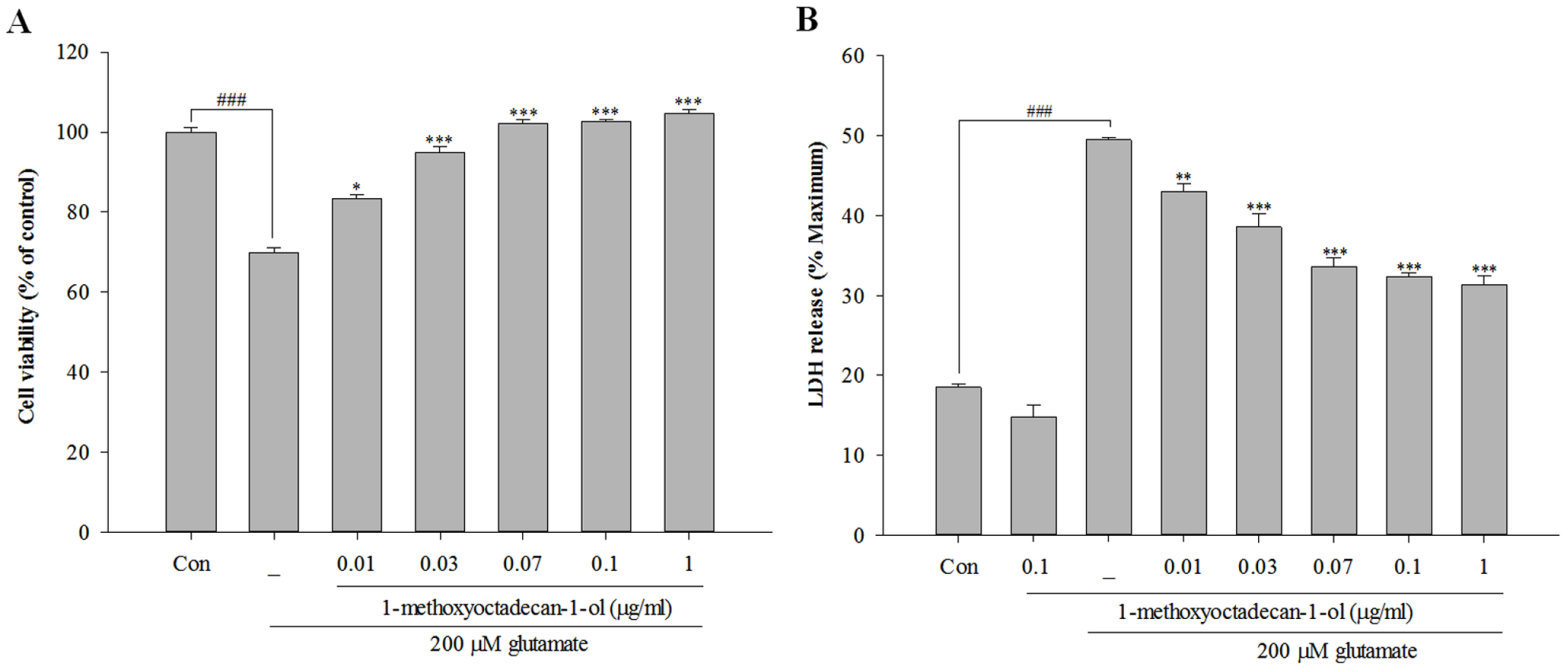
Concentration-dependent protective effects of 1-methoxyoctadecan-1-ol against glutamate-induced cell death in cultured cortical neurons. Cell viability and toxicity were determined by MTT (A) and LDH assay (B). Cortical neurons were pretreated with 0.01, 0.03, 0.07, 0.1, and 1 µg/ml of 1-methoxyoctadecan-1-ol for 24 h, followed by exposure to 200 µM glutamate for 6 h. ^###^ *P*<0.001 *vs*. control; *P<0.05, **P<0.01 and ***P<0.001 *vs*. treatment with glutamate alone. All data are represented as the mean±SEM of three independent experiments.

Neurons were pretreated with different concentrations of 1-methoxyoctadecan-1-ol (0.01–1 µg/ml) for 24 h and then exposed to 200 µM glutamate for 6 h. As shown in Fig. 2A, exposure of cortical neurons to glutamate resulted in reduced cell viability of approximately 30.2%, compared with the control. However, pretreatment with 1-methoxyoctadecan-1-ol resulted in significantly reduced glutamate-induced toxicity over a concentration range of 0.01 to 1 µg/ml. These findings were further verified by the LDH assay; as shown in Fig. 2B, pretreatment with 1-methoxyoctadecan-1-ol resulted in a marked decrease of glutamate-induced LDH release in a dose-dependent manner, compared with glutamate stimulation alone. Based on these results, we suggest that pretreatment with 1-methoxyoctadecan-1-ol can exert a potent neuroprotective effect against glutamate-induced toxicity.

### 1-methoxyoctadecan-1-ol Treatment Inhibits Glutamate-induced Apoptosis

**Figure 3:**
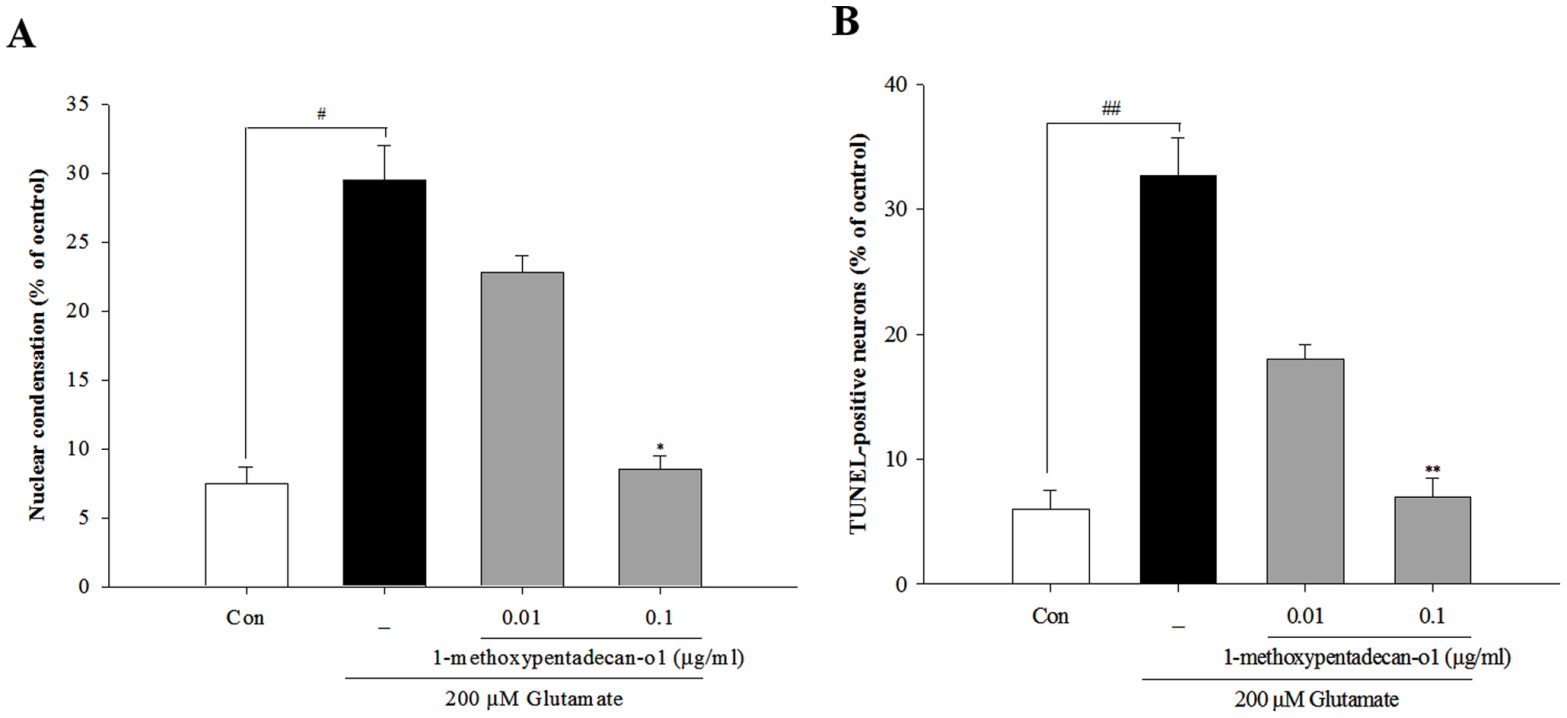
Effects of 1-methoxyoctadecan-1-ol on glutamate-induced apoptosis in cultured cortical neurons. Cortical neurons were pretreated with 1-methoxyoctadecan-1-ol (0.01 and 0.1 µg/ml) for 24 h, followed by exposure to 200 µM glutamate for 6 h. Quantitative analysis of the histograms for Hoechst 33342 (A) and TUNEL staining (B). ^#^ *P*<0.05 and ^##^ *P*<0.01 *vs*. control; *P<0.05, **P<0.01 *vs*. treatment with glutamate alone. Data are represented as the mean±SEM of three independent experiments.

**Figure 4:**
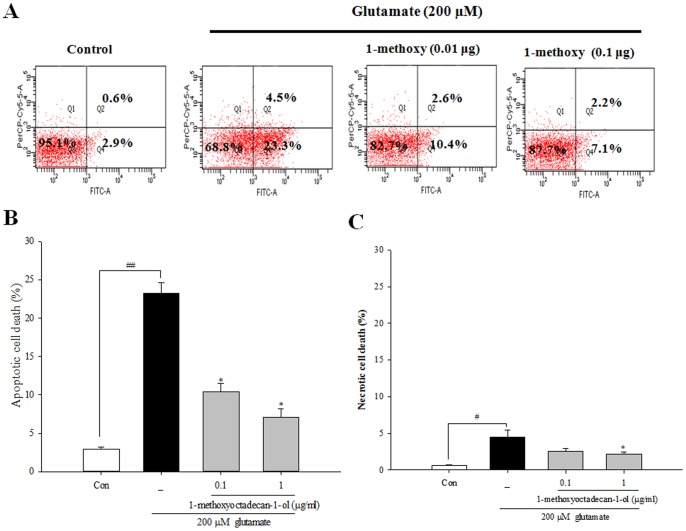
Flow cytometry analysis for neuronal death. Cortical neurons were pretreated with 1-methoxyoctadecan-1-ol (0.01 and 0.1 µg/ml) for 24 h, followed by exposure to 200 µM glutamate for 6 h. Cells were harvested and stained with Annexin V-FITC/PI, as described under methods and analyzed using flow cytometry. Annexin V^+^PI^−^ cells indicate early apoptotic cells, whereas Annexin V^+^PI^+^ cells are late apoptotic cells. The estimates (%) of gated cells in different compartments are given for each dot blot. Representative flow cytometry analysis scatter-grams of Annexin V/PI staining (A) and quantitative analysis of the histograms (B and C).^ #^ *P*<0.05 and ^##^ *P*<0.01 *vs*. control; *P<0.05 *vs*. treatment with glutamate alone. Data are represented as the mean±SEM of three independent experiments.

Exposure of neurons to excitotoxic levels of glutamate can result in programmed cell death that shares the features of both necrosis and apoptosis [19]. Nuclear morphological approaches were used to examine the question of whether 1-methoxyoctadecan-1-ol could inhibit glutamate-induced apoptosis/necrosis. In Hoechst 33342 staining, glutamate treated cortical neurons showed marked apoptotic features, compared to control, however, pretreatment of cortical neurons with 1-methoxyoctadecan-1-ol resulted in significant blockade of apoptotic neurons at the concentrations of 0.01 and 0.1 µg/ml (Fig. 3A). TUNEL-positive neurons showed an increase, compared to control; however, pretreatment with 1-methoxyoctadecan-1-ol resulted in a marked decrease in these positive neurons (Fig. 3B). To confirm the anti-apoptotic/necrosis effect, we used flow cytometry with annexin V/PI staining. As shown in Fig. 4, after exposure to glutamate, cortical neurons were likely to undergo mostly apoptotic cell death rather than necrotic death. Pretreatment with 1-methoxyoctadecan-1-ol resulted in a marked decrease in the apoptotic population (Fig. 4B). These results suggest that pretreatment with 1-methoxyoctadecan-1-ol exerts a neuroprotective effect by abrogating glutamate-induced apoptosis.

### 1-methoxyoctadecan-1-ol Preferentially Attenuates Glutamate-evoked NMDAR GluN2B Subunit Phosphorylation

**Figure 5:**
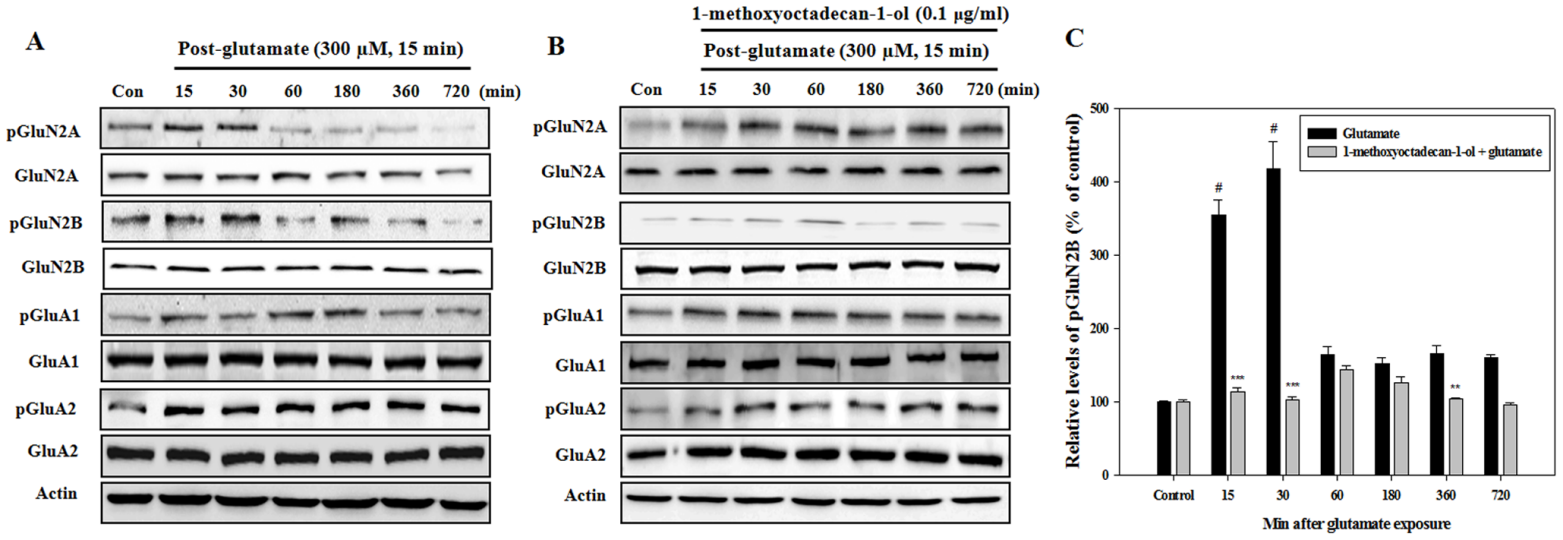
Time-course effects of 1-methoxyoctadecan-1-ol on NMDAR and AMPAR subunit after glutamate exposure. Cortical neurons were treated with glutamate (300 µM, 15 min) (A) and pretreatment with 1-methoxyoctadecan-1-ol (0.1 µg/ml, 24 h) followed by exposure to glutamate (B), then both were maintained in the original medium for the specified time. Densitometric analysis (C) showed that 1-methoxyoctadecan-1-ol treatment significantly decreased the phosphorylation of the NMDAR pGluN2B subunit. Equal amounts of proteins in each sample were subjected to Western blot assays using the indicated antibodies. Equal protein loading was confirmed by actin expression. ^#^ *P*<0.05 *vs*. control; **P<0.01 and ***P<0.001 *vs*. treatment with glutamate alone. Data are represented as the mean±SEM of three independent experiments.

In pathological conditions including massive glutamate release, over-activation of ionotropic glutamate receptors plays a critical role in neuroexcitotoxicity [9], [20]. To determine whether 1-methoxyoctadecan-1-ol could modulate glutamate-mediated activation of these receptors, cortical neurons were treated with 300 µM glutamate for 15 min, and Western blot analysis was performed. As shown in Fig. 5A, following exposure to glutamate alone, cortical neurons showed a sustained phosphorylation of α-amino-3-hydroxy-5-methyl-4-isoxazole propionic acid receptor (AMPAR) GluA1 and GluA2 subunit throughout the experiment, however, the NMDAR GluN2A and GluN2B subunits showed a rapid increase in expression of phosphorylation at early time points. Pretreatment with 1-methoxyoctadecan-1-ol resulted in a marked selective decrease in glutamate-stimulated phosphorylation of the NMDAR GluN2B subunit. But slight increase of phosphorylated NMDAR GluN2A subunit was observed at late phase of experiment (Fig. 5B). When we performed a histogram analysis, phosphorylation of the NMDAR GluN2B subunit was significantly arrested by 1-methoxyoctadecan-1-ol treatment throughout experiment (Fig. 5C). These results suggest that activation of the NMDAR GluN2B subunit could play a dominant role in mediating the toxic signal under our experimental conditions, which were down regulated by 1-methoxyoctadecan-1-ol pretreatment.

### 1-methoxyoctadecan-1-ol Down-regulates Calpain Activation, STEP Cleavage, and P38 MAPK Activation caused by Glutamate

**Figure 6:**
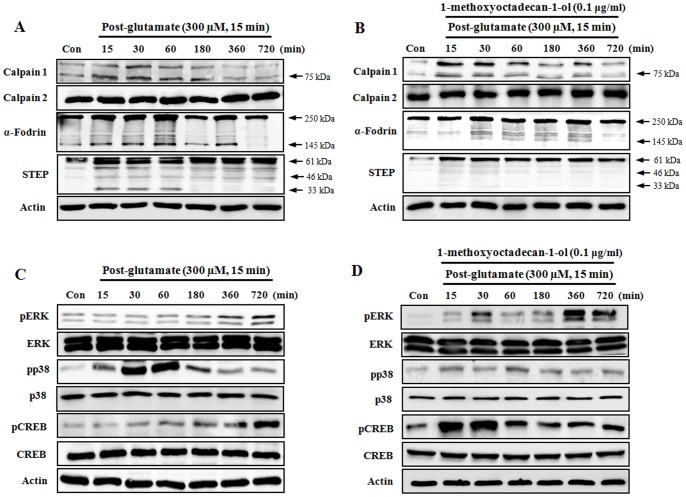
Time-course effects of 1-methoxyoctadecan-1-ol on calpain activation, STEP cleavage, and p38 MAPK phosphorylation after glutamate exposure. Cortical neurons were treated with glutamate (300 µM, 15 min) (A and C) and pretreatment with 1-methoxyoctadecan-1-ol (0.1 µg/ml, 24 h) followed by exposure to glutamate (B and D) and both were maintained in the original medium for the specified time. Equal amounts of proteins and each sample were subjected to Western blot assays using the indicated antibodies. Equal protein loading was confirmed by actin expression.

Recent reports have suggested that NMDAR-mediated excitotoxicity may trigger calpain activity, in which calpains can also act to cleave a variety of proteins, including STEP, which follows excitotoxic neuronal injury [10], [11], [16]. Therefore, we attempted to determine whether 1-methoxyoctadecan-1-ol could inhibit calpain activation and STEP cleavage caused by glutamate. As shown in Fig. 6A, after exposure to glutamate alone, cortical neurons showed a rapid increase in the active form of calpain1 (∼75 kDa), subsequently resulting in a marked increase in a breakdown product of fodrin (∼145/150 kDa), indicative of cleavage by activated calpain. However, pretreatment with 1-methoxyoctadecan-1-ol resulted in significantly reduced calpain1 activity and also reduced the accumulation of fodrin breakdown product caused by glutamate (Fig. 6B). Following excitotoxic insult, STEP, a substrate for Ca^2+^-dependent calpain, is rapidly cleaved to a smaller isoform, STEP_33_ [16], [21]. Pretreatment with 1-methoxyoctadecan-1-ol resulted in significantly blocked production of STEP_33_ caused by glutamate (Figs. 6A and 6B). Our results indicate that the neuroprotective effects of 1-methoxyoctadecan-1-ol could, at least in part, be attributed to down-regulation of calpain activation and STEP cleavage caused by glutamate.

Finally, we attempted to determine whether pretreatment with 1-methoxyoctadecan-1-ol can regulate ERK1/2 and p38 MAPK signaling because these kinases are a potential substrate of STEP [12], [22], [23]. As shown in Figure 6C, after exposure to glutamate alone, cortical neurons showed a significant increase in phosphorylation of p38 MAPK within 0.5–1 h after glutamate exposure, however, levels of both phosphorylated ERK1/2 and CREB were shown at a basal level, except during the late phase of the experiment. Pretreatment with 1-methoxyoctadecan-1-ol resulted in a significant decrease in the levels of phosphorylated p38 MAPK with increase of CERB phosphorylation. The phosphorylation of ERK showed a transient increase at 30 min after glutamate exposure (Fig. 6D). These results support that the idea that inhibition of calpain-mediated STEP cleavage, and subsequent alleviation of p38 MAPK activation is involved in the neuroprotective mechanisms of 1-methoxyoctadecan-1-ol.

### Inhibition of P38 MAPK Signaling Pathway Plays a Critical Role in 1-methoxyoctadecan-1-ol-mediated Neuroprotection

**Figure 7:**
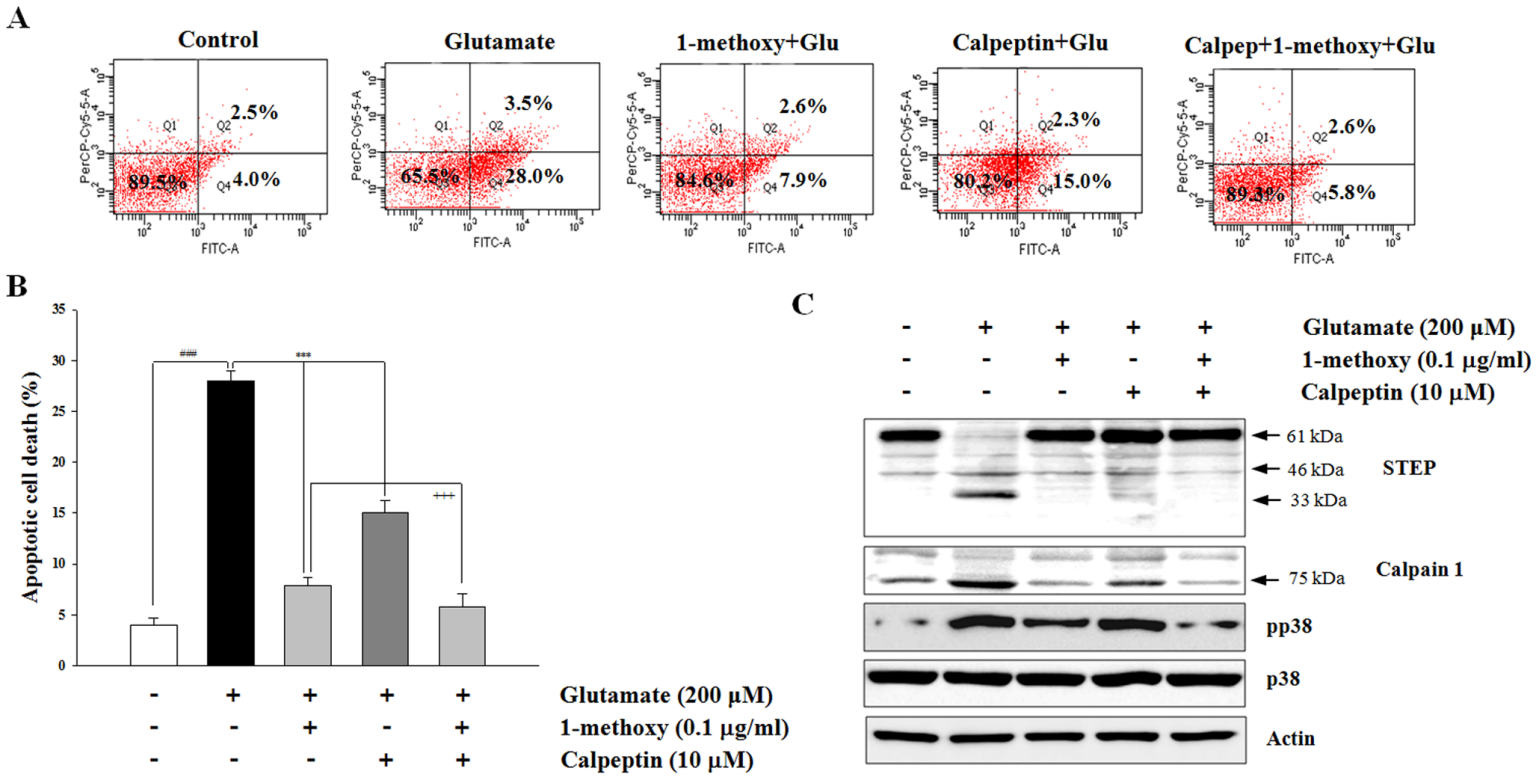
Effects of calpeptin on neuroprotection of 1-methoxyoctadecan-1-ol. Cortical neurons were pretreated with calpeptin (10 µM) for 1 h and subsequently treated with 1-methoxyoctadecan-1-ol (0.1 µg/ml) for 24 h, followed by exposure to 200 µM glutamate for 6 h. Representative flow cytometry analysis scatter-grams of Annexin V/PI staining (A) and its quantitative analysis of the histograms (B). Cell lysates were subjected to Western blot assays using calpain1, STEP, and p38 MAPK (C). Equal protein loading was confirmed by actin expression.^ ###^ *P*<0.001 *vs*. control; ***P<0.001 *vs*. treatment with glutamate alone; ^+++^ *P*<0.001 *vs*. 1-methoxyoctadecan-1-ol treatment with glutamate. Data are represented as the mean±SEM of three independent experiments.

**Figure 8:**
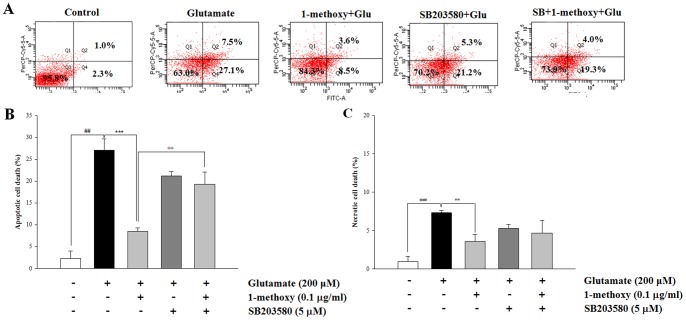
Effects of SB203580 on neuroprotection of 1-methoxyoctadecan-1-ol. Cortical neurons were pretreated with SB203580 (5 µM) for 30 min and subsequently treated with 1-methoxyoctadecan-1-ol (0.1 µg/ml) for 24 h, followed by exposure to 200 µM glutamate for 6 h. Representative flow cytometry analysis scatter-grams of Annexin V/PI staining (A) and quantitative analysis of the histograms (B–C).^ ##^ *P*<0.01, ^###^ *P*<0.001 *vs*. control; **P<0.01, ***P<0.001 *vs*. treatment with glutamate alone; ^++^ *P*<0.01 *vs*. 1-methoxyoctadecan-1-ol treatment with glutamate. Data are represented as the mean±SEM of three independent experiments.

We performed inhibitor studies to confirm that calpain and its downstream pathway is a major target for neuroprotectve effects. Neurons were pretreated with calpeptin (10 µM) or SB203580 (5 µM) for 30 min before addition of 1-methoxyoctadecan-1-ol and then exposed to glutamate for 6 h. As shown in Figs. 7A and B, treatment with calpain1 inhibitor calpeptin alone resulted in a marked decrease of glutamate-induced apoptosis. Further, co-treatment with calpeptin and 1-methoxyoctadecan-1-ol resulted in a robust neuroprotection, compared to each treatment alone. When we checked calpain activation, STEP cleavage, and p38 MAPK activation again, each 1-methoxyoctadecan-1-ol and calpeptin showed inhibitory effects. Boosting the effects of co-treatment also contributed to complete blockade of these expressions (Fig. 7C). We then applied p38 MAPK inhibitor SB203580, downstream of calapin/STEP. As shown in Fig. 8, treatment with SB203580 alone resulted in partially reduced glutamate-induced apoptosis. However, co-treatment with 1-methoxyoctadecan-1-ol and SB203580 failed to result in a marked neuroprotective effect; therefore, SB203580 may have abolished the neuroprotective action of 1-methoxyoctadecan-1-ol. These results provide strong evidence that inhibition of the p38 MAPK pathway via partial calpain-mediated STEP cleavage may play a critical role in 1-methoxyoctadecan-1-ol-mediated neuroprotection.

### Neuroprotective Effects of 1-methoxyoctadecan-1-ol against Photothrombotic Ischemic Injury in Mice

**Figure 9:**
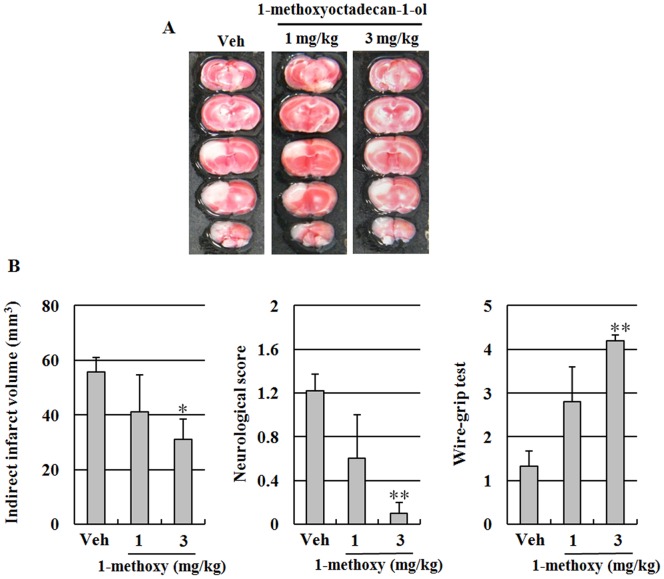
Effects of 1-methoxyoctadecan-1-ol on infarct volume, neurological evaluation, and wire-grip test in a photothrombotic ischemic model. Mice received intraperitoneal administration of DMSO or 1, 3/kg of 1-methoxyoctadecan-1-ol at 30 min before the ischemic insult. Representative photographs of coronal brain sections stained with TTC in vehicle (Veh)- and 1-methoxyoctadecan-1-ol-treated mice (A). White indicates the infarct area. Quantification of the infarct volume, neurological score, and wire-grip test (B). *P<0.05, **P<0.01 *vs*. vehicle group. Data are expressed as mean±SEM of three separate experiments.

**Figure 10:**
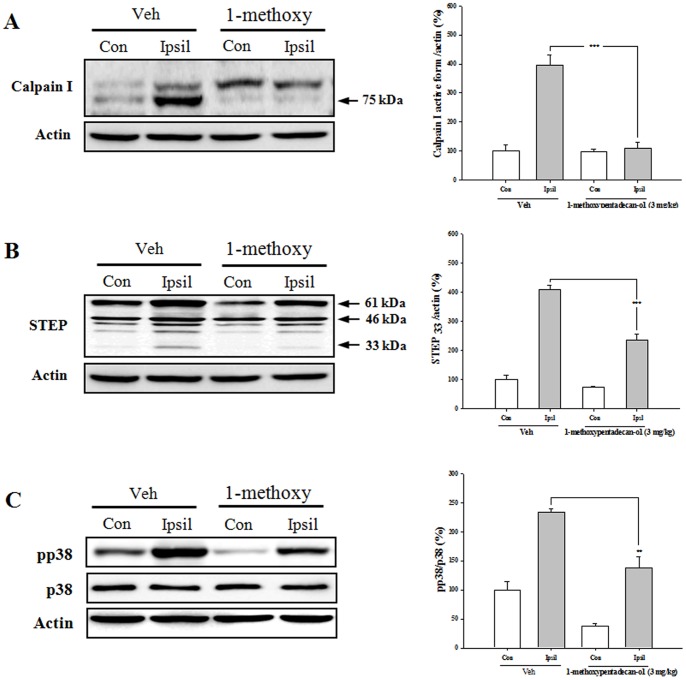
Effects of 1-methoxyoctadecan-1-ol on calpain activation, STEP cleavage and p38 MAPK phosphorylation. The significant calpain1 activation (A), STEP cleavage (B) and p38 MAPK phosphorylation (C) were shown in the ipsilateral (Ipsil) cerebral hemisphere of photothrombotic ischemic mice compared with the contralateral (Con). **P<0.01, ***P<0.001, *vs*. vehicle group, Data are expressed as mean±SEM of three separate experiments.

To investigate the potential neuroprotective efficacy of 1-methoxyoctadecan-1-ol *in vivo*, we tested this agent in a photothrombotic ischemic mouse model. 1-methoxyoctadecan-1-ol (1 and 3 mg/kg, i.p.) was administered 30 min before ischemic insults. Pretreatment with 1-methoxyoctadecan-1-ol resulted in significantly decreased cerebral infarct volume and improved neurological function at both concentrations, as compared to the vehicle group (Figs. 9A and B). These results suggested that 1-methoxyoctadecan-1-ol has a promising protective effect even under focal cerebral ischemia. Next, we attempted to determine whether inhibition of calpain-mediated STEP cleavage and p38 MAPK activation play a critical role in the outcomes of a cerebral ischemia model. As shown in Fig. 10, results of Western blot clearly demonstrated that 1-methoxyoctadecan-1-ol induced a significant reduction of the ischemia-induced active form of calpain1 with blocked production of STEP_33_. In addition, treatment with 1-methoxyoctadecan-1-ol resulted in significantly blocked ischemia-induced phosphorylation of p38 MAPK. These results demonstrated that 1-methoxyoctadecan-1-ol may also prevent ischemia-induced neuronal damage through inhibition of calpain-mediated STEP cleavage and p38 MAPK activation.

## Discussion

In some East Asian countries, the hooks and stems of *Uncaria* plants, such as *U. rhynchophylla*, *U. sinensis, U. macrophylla*, and *U. sessilifructus* are known to provide relief from various nervous related symptoms [3], [24]. Earlier investigations have established that several alkaloids and phenolic compounds from *Uncaria* species have availability for use as a pharmacological medicine against nervous disorders [1], [4], [18].

Because hexane extract from *U. sinensis* exhibits anti-apoptotic effects [6], this extract may be an attractive candidate for use as a therapeutic compound for treatment of neurodegenerative diseases associated with excitotoxicity. We isolated a novel neuroprotectant, 1-methoxyoctadecan-1-ol, from *U. sinensis* (Fig. 1), and explored the molecular mechanism underlying the neuroprotective effects of 1-methoxyoctadecan-1-ol on glutamate-mediated excitotoxic signaling and applicability for cerebral ischemia.

First, we observed anti-apoptotic properties of methoxyoctadecan-1-ol against sustained glutamate toxicity in primary cortical neurons. Pretreatment with 1-methoxyoctadecan-1-ol resulted in concentration-dependent inhibition of glutamate-induced toxicity (Fig. 2) and prevention more specifically at a very lower dose, compared with our previous studies [6]. Exposure to excitotoxic levels of glutamate induces two distinct types of programmed cell death, sharing both necrosis and apoptosis [19]. Our results demonstrated that 1-methoxyoctadecan-1-ol had strong neuroprotective effects through inhibition of glutamate-induced apoptosis, rather than necrosis (Figs. 3 and 4). We then examined signaling pathways underlying neuroprotective mechanisms, focusing on excitotoxic neuronal death.

Excitotoxicity, over-activation of glutamate receptors by marked increase of extracellular glutamate levels, plays an important role in many acute and chronic diseases, including ischemic stroke, epilepsy, Alzheimer’s, and Parkinson’s diseases [16], [25]. Excessive Ca^2+^ influx through excessive activation of NMDAR and AMPAR commonly contributes to Ca^2+^-dependent neuronal death, disrupting normal intracellular signaling [16], [26]–[28].

According to our results (Fig. 5), glutamate-treated cortical cells showed a sustained AMPAR and an early increase of NMDAR phosphorylation. Because excitotoxicity shows whthin a relatively short period of time in ischemic stroke, we focused on the changes of ionotropic glutamate receptor during the early phase of the experiment.

Glutamate-induced phosphorylation of GluN2B-NMDAR at the Ser1303 site was selectively decreased by 1-methoxyoctadecan-1-ol treatment, which suggests that the GluN2B subunit could play a dominant role in arrest of neuronal death. Phosphorylation of the NMDAR GluN2B subunit at the 1303 site leads to enhancement of channel conductance, which may lead to ischemic neuronal death [29].

Both neuroprotective and neurotoxic functions may present via synaptic and extrasynaptic NMDAR, respectively. GluN2B-MDAR is isolated primarily at the extrasynaptic site, while GluN2A-NMDAR at the synaptic site [30]. The NMDAR GluN2B subunit is generally thought to be involved in triggering intracellular cascades that lead to neuronal apoptosis following an excitotoxic insult [10], [29], [30].

Synaptic stimulation of NMDAR leads to ubiquitination and degradation of STEP_61_, concomitant with the Ras-ERK-CREB pathway and translation of pro-survival protein BDNF [16], [31]–[33]. In contrast, extrasynaptic stimulation of NMDAR promotes selective calpain-mediated proteolysis of STEP_61_, leading to production of the truncated cleavage product, STEP_33_, and activation of p38 MAPK invokes important proapoptotic proteins to excitotoxic neuronal injury [10], [12], [16], [33]. Therefore, differential NMDAR stimulation for degradation of STEP_61_ and subsequent activation of ERK1/2 or p38 MAPK is a valid target for development of neuroprotective therapy [16].

Our results are consistent with general excitotoxic neuronal injury, in which calpain-mediated proteolytic cleavage of STEP_61_ was clearly observed following exposure to glutamate. Treatment with glutamate alone induced up-regulated activation of calpain1, and also led to subsequent production of STEP_33_. Detectable 145 kDa protein fragments of α-fodrin were also demonstrated, indicating cleavage of this protein by activated calpain (Fig. 6A). However, pre-treatment with 1-methoxyoctadecan-1-ol resulted in selectively attenuated activation of calpain1 with decreased α-fodrin fragment, and, subsequently, reduced production of STEP_33_ (Fig. 6B).

Excitotoxic levels of the neurotransmitter glutamate present higher levels of Ca^2+^ influx, which occurs by GluN2B-NMDAR rather than an initial rapid rise by GluN2A, and Ca^2+^-activated calpains mediate, at least in part, glutamate-induced neurotoxicity [34], [35]. The GluN2B-specific antagonist, ifenprodil, attenuates STEP_33_ production, in which this receptor may mediate cleavage of STEP_61_ [10], [16]. Our results suggest that calpain1 may be activated by significant increases in cytosolic Ca^2+^ via GluN2B-NMDAR and that 1-methoxyoctadecan-1-ol can selectively attenuate these activations involving in triggering intracellular cascades for phosphorylation.

STEP exists as two major alternatively spliced isoforms, membrane associated STEP_61_ and cytosolic STEP_46_, and dephosphorylates its substances, including ERK1/2, p38 MAPK, the Src family tyrosine kinase Fyn, and the NMDAR GluN2B subunit at regulatory tyrosine residues, which leads to inactivation of these neuronal enzymes [16], [36], [37]. STEP serves as a modulator of NMDAR-dependent neuronal injury. If GluN2B-NMDAR stimulation is sustained, these stimuli cause significant degradation of active STEP, leading to neuronal injury through its regulation of p38 MAPK [10].

Our western blot results showed that pretreatment with 1-methoxyoctadecan-1-ol induced rapid attenuation of glutamate-stimulated p38 MAPK activation along with early ERK1/2 phosphorylation and transcription factor CREB (Figs. 6C and D). The selective inhibition of 1-methoxyoctadecan-1-ol for cleavage of STEP61 by calpain showed clearly involvement of p38 MAPK. NMDAR stimulation may leads to ERK1/2 activation to promote neuronal survival, and phosphorylation of STEP61 at Ser221 is required for ubiquitination [12]. But there were no significant changes of STEP phosphoryaltion with 1-methoxyoctadecan-1-ol treatment or not (data not shown). These findings demonstrated that inhibition of p38 MAPK plays a critical role in the neuroprotective actions of 1-methoxyoctadecan-1-ol. To confirm our proposed signaling, we performed inhibitor studies using calpain1 inhibitor, because calpain is one of the initial triggers in cascade events associated with p38 MAPK-mediated cell death.

Flow cytometric results of 1-methoxyoctadecan-1-ol and calpeptin co-treatment indicated a synergistic protective effect (Fig. 7), suggesting that methoxyoctadecan-1-ol may prevent excitoxic neuronal death via a different pathway, not calpain-mediated STEP cleavage. p38 MAPK plays an integral role in caspase activation and mitochondrial dysfunction by Bcl-2 family [38], [39] and blocking via the PI3K/Akt signaling pathway with downstream effector Bcl-2 [40], [41]. Our previous results showing that hexane extract from *U. sinensis* enhanced Bcl-2 expression with alleviation of caspase activation [6] may suggest possible involvement of another pathway. However, Western blot analysis showed that co-treatment resulted in complete blockade of all activated calpain, STEP cleavage, and p38 phosphorylation, in which methoxyoctadecan-1-ol exerted neuroprotective effects similar to those of calpeptin. Further, pretreatment with p38 MAPK inhibitor, SB203580, resulted in arrest of the neuroprotective effects of methoxyoctadecan-1-ol, supporting that inhibition of p38 MAPK may be a main critical pathway in 1-methoxyoctadecan-1-ol-mediated neuroprotection (Fig. 8).

The ischemic animal model shows that STEP is unilaterally down regulated in the stroke hemisphere, STEP_61_ is cleaved into a novel species, STEP_33_, by calpain [21], and initiates cell death pathways by activation of p38 MAPK [16]. This calpain-mediated STEP proteolysis reflects an important outcome of ischemic brain injury, and degradation of STEP plays a critical role in p38 MAPK activation [10], [21].

Protective effect of methoxyoctadecan-1-ol is further supported by an *in vivo* photothrombotic ischemic model (Fig. 9). Consistent with previous findings, Western blot data also demonstrated that 1-methoxyoctadecan-1-ol effectively down-regulates calpain1 activity and cleavage of STEP_33_ with significantly decreased p38 MAPK phosphorylation in the ischemic hemisphere (Fig. 10). These results suggest that 1-methoxyoctadecan-1-ol could be an attractive candidate for use in the therapeutic approach to ischemic injury.

Consequently, our studies demonstrated that 1-methoxyoctadecan-1-ol exerts its neuroprotection against glutamate-induced apoptosis in primary cultured neurons by down-regulation of GluN2B-NMDAR and calpain activation with subsequent reduction in STEP cleavage and p38 MAPK activation. 1-methoxyoctadecan-1-ol can also contribute to the promising beneficial effects against ischemic brain injury, and this novel compound may be a highly effective therapeutic agent in treatment of neurodegenerative diseases associated with excitotoxic or ischemic insults.
